# In COS Cells Vpu Can Both Stabilize Tetherin Expression and Counteract Its Antiviral Activity

**DOI:** 10.1371/journal.pone.0111628

**Published:** 2014-10-31

**Authors:** Abdul A. Waheed, Nishani D. Kuruppu, Kathryn L. Felton, Darren D’Souza, Eric O. Freed

**Affiliations:** Virus-Cell Interaction Section, HIV Drug Resistance Program, NCI-Frederick, Frederick, Maryland, United States of America; Helmholtz Zentrum Muenchen - German Research Center for Environmental Health, Germany

## Abstract

The interferon-inducible cellular protein tetherin (CD317/BST-2) inhibits the release of a broad range of enveloped viruses. The HIV-1 accessory protein Vpu enhances virus particle release by counteracting this host restriction factor. While the antagonism of human tetherin by Vpu has been associated with both proteasomal and lysosomal degradation, the link between Vpu-mediated tetherin degradation and the ability of Vpu to counteract the antiviral activity of tetherin remains poorly understood. Here, we show that human tetherin is expressed at low levels in African green monkey kidney (COS) cells. However, Vpu markedly increases tetherin expression in this cell line, apparently by sequestering it in an internal compartment that bears lysosomal markers. This stabilization of tetherin by Vpu requires the transmembrane sequence of human tetherin. Although Vpu stabilizes human tetherin in COS cells, it still counteracts the ability of tetherin to suppress virus release. The enhancement of virus release by Vpu in COS cells is associated with a modest reduction in cell-surface tetherin expression, even though the overall expression of tetherin is higher in the presence of Vpu. This study demonstrates that COS cells provide a model system in which Vpu-mediated enhancement of HIV-1 release is uncoupled from Vpu-mediated tetherin degradation.

## Introduction

Mammalian cells have evolved a variety of strategies to prevent virus replication. These include constitutive or inducible expression of a number of restriction factors that interfere with different stages of the virus replication cycle. Many of these restriction factors are induced by type-I interferon (IFN) as a component of the innate immune system. Host cell restriction factors target the incoming virus, act at the level of transcription, or disrupt late stages of the replication cycle. Tetherin was identified as an IFNα-inducible restriction factor that tethers mature viral particles to the infected cell surface [1], [2]. While the physiological function of tetherin is not clearly understood, it is expressed constitutively in terminally differentiated B cells, monocytes, primary bone marrow stromal cells, and plasmacytoid dendritic cells [3]–[6]. Tetherin is a type-II integral membrane protein with an unusual topology: it bears an N-terminal cytoplasmic domain, followed by a transmembrane (TM) domain, a coiled-coil, and a putative C-terminal glycosylphosphatidylinositol (GPI) anchor [7]. Membrane anchors at both N- and C-terminal regions of tetherin are required for antiviral activity [1], [8], [9]. Tetherin restricts the release of a broad spectrum of enveloped viruses, including not only HIV-1 but also other retroviruses, filoviruses, arenaviruses, and herperviruses [10]–[12].

Lentiviruses have developed several distinct strategies for evading the antiviral activity of tetherin. HIV-2 and some strains of simian immunodeficiency virus (e.g., SIVtan) express an Env glycoprotein that acts as a tetherin antagonist by inducing its sequestration in an intracellular compartment that bears markers for the trans-Golgi network (TGN) [13]–[15]. Serra-Moreno et al. reported that a Nef-deleted strain of SIV adapts to overcome rhesus tetherin by acquiring changes in the cytoplasmic tail of Env [16]. Other strains of SIV antagonize simian but not human tetherin through their Nef proteins [17]–[19]. The herpes simplex virus 1 (HSV-1) glycoprotein M, the Env proteins of equine infectious anemia virus (EIAV), human endogenous retrovirus type K (HERV-K), and feline immunodeficiency virus (FIV), and the chikungunya virus non-structural protein 1 (nsP1) antagonize tetherin restriction [20]–[24]. HIV-1 Vpu counteracts human, chimpanzee, and gorilla tetherin but is relatively inactive against tetherin from other non-human primates or from non-primate species (e.g., the mouse) [17], [18], [25]–[27]. Nonetheless, Shengai et al. reported that Vpu from some simian-human immunodeficiency virus (SHIV) chimeras is capable of antagonizing macaque tetherin [28]. SIVcpz, the chimpanzee precursor to HIV-1 [29], encodes a Nef protein that is able to counteract chimpanzee but not human tetherin. Following transfer to humans, group M HIV-1 (the main pathogenic group of HIV-1 responsible for the AIDS epidemic) acquired the ability to antagonize human tetherin through its Vpu protein [18]. In contrast, Vpu from the less-pathogenic HIV-1 group O strains has limited capacity to downregulate tetherin [18]. Thus, the ability of Vpu to counteract human tetherin may have played a significant role in the current AIDS pandemic.

The mechanism by which HIV-1 Vpu counteracts the antiviral activity of human tetherin remains to be elucidated (for reviews, [30], [31]). A number of diverse findings have been reported. Upon Vpu expression, previous studies have observed reduced cell-surface tetherin levels with no effect on overall expression [2], [32] or a reduction in overall tetherin levels via lysosomal [33], [34] or proteasomal [27], [35], [36] degradation pathways. Immuno-electron microscopy analysis indicated that Vpu shifts the localization of tetherin from the plasma membrane (PM) to early and recycling endosomes [37]. Several studies have demonstrated Vpu-mediated antagonism of tetherin in the absence of significantly reduced expression [1], [38], [39]. It has also been reported that Vpu induces the sequestration of tetherin in a perinuclear compartment, consistent with Vpu interfering with the trafficking of tetherin from the TGN to the PM [40]. Vpu has been reported to disrupt both the transport of newly synthesized tetherin to the PM and the recycling of internalized tetherin back to the cell surface without affecting rates of tetherin internalization [40]–[42]. Rollason et al. reported that Vpu translocates endogenous tetherin out of lipid rafts and induces its internalization into endosomes and degradation in lysosomes [43].

While the main function of tetherin is to restrict HIV-1 release, it may also affect cell-cell virus transfer and particle infectivity. Data have been published supporting a role for tetherin in enhancing [44] or restricting [45]–[47] cell-cell spread of HIV-1. It has also been reported that tetherin expression can impair specific HIV-1 particle infectivity [48], whereas others observed a similar reduction in particle release and infectivity for Vpu(−) compared with Vpu(+) virus produced in (tetherin-expressing) HeLa cells [37]. Recently, it was reported that anti-tetherin activity of Vpu protects HIV-1-infected cells from antibody-dependent cell-mediated cytotoxicity (ADCC) [49]–[51].

In this study, we show that Vpu-mediated degradation of tetherin occurs by both proteasomal and lysosomal pathways, and that the absence of Vpu antagonism of non-human tetherin is associated with a lack of tetherin degradation. We observe that human tetherin is poorly expressed in agm kidney (COS) cells and that Vpu markedly enhances expression of human tetherin in this cell line. The stabilization of tetherin by Vpu is specific to human tetherin, and requires the tetherin TM sequence implicated in Vpu–tetherin binding. Although the cellular expression of tetherin is enhanced by Vpu in COS cells, its surface expression and virus-restricting capacity are reduced by Vpu. These observations confirm that Vpu-mediated degradation of tetherin and antagonism of tetherin restriction are two separable functions of Vpu [38], [39] and demonstrate that the consequences of the Vpu–tetherin interaction are strongly cell type dependent.

## Materials and Methods

### Plasmids, antibodies, and chemicals

The full-length infectious HIV-1 molecular clone pNL4-3 and the Vpu-defective counterparts pNL4-3delVpu and pNL4-3Udel were described previously [53]–[55]. The codon-optimized plasmid pcDNA-Vphu was used for expressing the Vpu protein [56]. pNL4-3delVpu, pNL4-3Udel, and pcDNA-Vphu were kindly provided by K. Strebel. The HA epitope-tagged tetherin expression vector, the deletion mutant derivatives delCT and delGPI, and the mouse, rh, agm, and chimeric tetherin constructs [hu(rhTM)] or agm [hu(agmTM)] [1], [25] were generously provided by P. Bieniasz. The fluorescently-tagged Gag expression plasmid containing a monomeric Eos fluorescent protein (mEosFP) was constructed by inserting the mEosFP coding region between the MA and CA domains of Gag using a strategy similar to that reported by Hubner et al. to construct Gag-iGFP [57]. Briefly, a synthetic viral protease cleavage sequence, SQNYPIVQ, followed by a polylinker containing BstBI and PacI restriction sites was introduced between the MA and CA domains of the HIV-1 Gag expression vector pNL4-3ΔPolΔEnv, in which the *pol* and *env* genes are deleted. A large portion of *pol* (nt 2429–4377) was removed by restriction digestion with BciI and NsiI, and a portion of *env* (nt 6530–7611) was deleted by restriction enzymes NsiI and BgiII. The fragments were ligated after treating with T4 DNA polymerase to create blunt ends. The mEosFP coding region was amplified from the pmEosFP2-C1 plasmid [58] (a kind gift from J. Lippincott-Schwartz) flanked by AclI (5′) and BsiEI (3′) restriction sites. This fragment was inserted into the BstBI and PacI restriction sites of the synthetic cleavage sequence to generate pNL4-3Gag-imEosFPΔPolΔEnv. Anti-HA and anti-tubulin antibodies, and concanamycin A, were purchased from Sigma (St. Louis, MO). Anti-Vpu [59], anti-human tetherin [39], and anti-HIV-1 Ig were obtained from the NIH AIDS Research and Reference Reagent Program. Anti-TGN46 was purchased from AbD Serotec (MorphoSys), anti-CD63 from Santa Cruz Biotechnology, and anti-LAMP-1 from BD Biosciences. MG132 was obtained from A.G. Scientific Inc, N-acetyl-leu-leu-norleucinal (ALLN) from Calbiochem, and bafilomycin from Tocris Bioscience.

### Cell culture, transfection and preparation of virus stocks

293T, COS, and Vero cells were obtained from American Type Culture Collection and maintained in Dulbecco’s modified Eagle’s medium (DMEM) containing 10% fetal bovine serum (FBS). One day after plating, the cells were transfected with the indicated plasmid DNA in the absence or presence of WT or mutant tetherin expression vectors using Lipofectamine 2000 (Invitrogen Corp. Carlsbad, CA) according to the manufacturer’s recommendations. Cells and virus were harvested 24 h posttransfection.

### Western blotting analysis

For immunoblot analyses, cells were washed with PBS, and then lysed in a buffer containing 50 mM Tris-HCl (pH 7.4), 150 mM NaCl, 1 mM EDTA, 0.5% Triton X-100, and protease inhibitor cocktail (Roche). Proteins were denatured by boiling in sample buffer and subjected to SDS-PAGE, transferred to PVDF membrane, and incubated with appropriate antibodies as described in the text. Membranes were then incubated with HRP-conjugated secondary antibodies, and chemiluminescence signal was detected by using Western Lightning Chemiluminescence Reagent Plus (PerkinElmer, Wellesley, MA). Quantification of the intensities of the protein bands was performed by using an Alpha Innotech Fluorchem SP imager (Alpha Innotech Inc., CA).

### Pulse-chase analysis of tetherin

293T and COS cells were transfected with the vector expressing HA-tagged human tetherin in the absence and presence of pcDNA-Vphu. One day post-transfections cells were pulse-labeled for 30 min with [35S]Met-Cys, after which the labeling medium was replaced with DMEM containing 10% FBS and cultured for 1, 2, or 4 h. Cells were harvested at each time point and solubilized in NP40 containing lysis buffer and immunoprecipitated with anti-HA antibodies and analyzed by SDS-PAGE followed by fluorography [60].

### Virus release assays

293T cells were cotransfected with pNL4-3 or pNL4-3delVpu and human or agm tetherin plasmids in the absence or presence pcDNA-Vphu. One day posttransfection, cells were metabolically labeled for 2 h with [35S]Met-Cys and virions were pelleted in an ultracentrifuge. Viral proteins in cell and virus lysates were immunoprecipitated with HIV-Ig and analyzed by SDS-PAGE followed by fluorography [60]. The virus release efficiency was calculated as the amount of virion-associated Gag as a fraction of total (cell plus virion-associated) Gag.

### Flow cytometry

293T and COS cell lines were transfected with human tetherin in the absence or presence of Vpu. Twenty-four hours after transfection, cells were harvested by adding a solution of 1 mM EDTA in PBS and washed twice in ice-cold 1% BSA-PBS. The cells were then incubated with polyclonal anti-tetherin antiserum or normal rabbit serum in 1% BSA-PBS for 1 h at 4°C. The cells were then washed three times in 1% BSA-PBS, and stained with Alexa-488-conjugated anti-rabbit IgG secondary antibody in 1% BSA-PBS for 1 h at 4°C. The cells were fixed in 1% paraformaldehyde after washing three times and analyzed with a Becton Dickinson FACS Calibur flow cytometer.

### Immunofluorescence microscopy

For microscopy studies, 293T and COS cells cultured in chamber slides were transfected with pNL4-3Gag-imEosFPΔPolΔEnv and human tetherin expression vector in the absence or presence of Vpu plasmid. 12, 18, or 24 h posttransfection, cells were rinsed with PBS and fixed in 3.7% paraformaldehyde in PBS for 30 min. The cells were rinsed with PBS, permeabilized with methanol at −20°C for 4 min, washed in PBS and incubated with 0.1 M glycine-PBS for 10 min to quench the remaining aldehyde residues. After blocking with 3% BSA-PBS for 30 min, cells were incubated with primary antibodies (specific for tetherin, HA, Vpu, TGN46, CD63, or LAMP-1) appropriately diluted in 3% BSA-PBS for 1 h. The cells were washed with PBS three times and then incubated with secondary antibody conjugated with either Texas Red or Alexa-488 diluted in 3% BSA-PBS. In co-staining experiments in which both antibodies were raised in mouse (HA, LAMP-1, CD63), the primary antibodies were directly labeled with either Zenon Alexa Flour 488 or Zenon Alexa Flour 594 mouse IgG1 labeling kit (Invitrogen). After washing with PBS three times, cells were mounted with Vectashield mounting media with DAPI (Vector Laboratories) and examined with a Delta-Vision RT deconvolution microscope. Colocalization was quantified by Pearson correlation coefficient (R) values using the softWoRx colocalization module. For surface staining of tetherin, cells were incubated with human tetherin antibody prior to fixation with formaldehyde.

## Results

### Vpu-mediated tetherin degradation in 293T cells is prevented by proteasome inhibitors and to a lesser extent by lysosomal inhibitors

To investigate the mechanism by which Vpu counteracts the antiviral activity of tetherin, we expressed HA-tagged tetherin in the absence or presence of Vpu in 293T cells, in which endogenous tetherin expression in undetectable [1]. As shown in Fig. S1A, steady-state levels of tetherin were significantly reduced by Vpu expressed from a CMV-driven expression vector. A similar reduction in tetherin levels was observed when Vpu was expressed from the full-length HIV-1 molecular clone pNL4-3 (Fig. S1A). No reduction in tetherin levels was observed when 293T cells were cotransfected with HA-tetherin and the Vpu-defective molecular clones, delVpu and Udel (Fig. S1A). These results demonstrate, consistent with some previous reports [27], [32], [34], [36], that Vpu reduces the steady-state levels of tetherin.

Both proteasomal and lysosomal routes have been proposed for Vpu-mediated tetherin degradation [27], [33]–[36]. To investigate through which pathway tetherin undergoes degradation, we used proteasomal (MG132 and ALLN) or lysosomal (bafilomycin and concanamycin) inhibitors to treat 293T cells expressing tetherin with or without Vpu. We observed that treating Vpu and tetherin-expressing cells with proteasomal inhibitors markedly enhanced the expression of the putative non-glycosylated and singly glycosylated forms (∼23–26 kDa), and the higher molecular weight (∼30–36 kDa), fully glycosylated forms, of tetherin (Fig. S1B). Treating tetherin-expressing cells with lysosomal inhibitors markedly increased the expression of the ∼26 kDa form, whereas the highly glycosylated forms of tetherin (∼30–36 kDa) were not recovered significantly (Fig. S1B). These results suggest that the Vpu-mediated degradation of tetherin in 293T cells proceeds via both proteasomal and lysosomal routes, and that different tetherin species appear to be degraded predominantly by one or the other pathway.

### Tetherin from mouse, rhesus macaque and African green monkey are not degraded by Vpu

**Figure 1:**
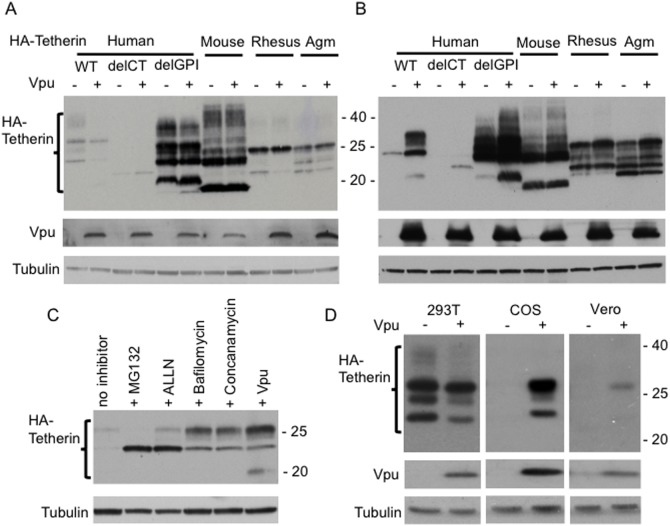
Vpu induces the degradation of human tetherin in 293T cells but stabilizes its expression in COS cells. 293T (A) or COS (B) cells were transfected with vectors expressing HA-tagged human, mouse, rhesus, and agm tetherins without or with Vpu expression vector at a tetherin:Vpu DNA ratio of 1∶5. Total transfected DNA was held constant with empty vector. Truncated human tetherins that lack the cytoplasmic tail (delCT) or putative GPI anchor (delGPI) were also used to identify the regions of tetherin required for Vpu-mediated degradation. One day posttransfection, cells were lysed and subjected to western blot analysis with the indicated antibodies. Vpu decreased tetherin expression by 3.1 fold in 293T cells whereas it increased tetherin expression by ∼5-fold in COS cells; levels of the delCT mutant were increased ∼3-fold in COS cells and 1.6 fold in 293T cells by Vpu. (C) COS cells were transfected with HA-tagged human tetherin expression vector and cell lysates were subjected to western blot analysis with anti-HA or anti-tubulin antibodies. Vpu was also co-expressed to compare the pattern of tetherin expression. Molecular mass markers are shown on the right (in kDa). In this experiment, proteasomal inhibitors (MG132 and ALLN) increased the expression of the ∼23 kDa tetherin species by 3.2 fold, whereas lysosomal inhibitors (bafilomycin and concanamycin) rescued the expression of the ∼26 kDa tetherin species by 2.6 fold and the ∼23 kDa tetherin species by 1.7 fold compared to the no inhibitor control. Co-expression of Vpu increased the expression of the ∼26 kDa tetherin species by 5.2 fold and the ∼23 kDa tetherin species by 1.7 fold. (D) 293T, COS, and Vero cells were transfected with HA-tagged human tetherin expression vector in the absence and presence of Vpu expression vector, and one-day posttransfection cells were lysed and subjected to Western blot analysis as above. Vpu reduced the expression of tetherin by 2.4 fold in 293T cells, whereas the levels were increased by ∼20-fold in COS cells and ∼4-fold in Vero cells. Similar results were obtained in an independent experiment.

Recent studies have demonstrated that tetherins from mouse, rhesus macaque (rh), and African green monkey (agm) inhibit HIV-1 release, but are not counteracted by Vpu [17], [25]–[27]. To investigate whether the absence of Vpu antagonism is due to the inability of Vpu to induce the degradation of these non-human tetherins, we expressed mouse, rh and agm tetherin in 293T cells in the absence or presence of Vpu and analyzed tetherin expression by western blot. As shown above, the expression of human tetherin is reduced considerably in the presence of Vpu. In contrast, and consistent with previous reports [17], [26], [27], Vpu has no effect on the expression levels of mouse, rh, or agm tetherin (Fig. 1A). Next, we investigated whether Vpu could also degrade human tetherin mutants that do not inhibit HIV-1 release. We expressed inactive human tetherin mutants that lack the cytoplasmic tail (delCT) or the putative GPI anchor (delGPI). We observed that Vpu has only minor effects on the expression of these mutants, demonstrating that Vpu-mediated degradation of human tetherin requires both the CT and the putative GPI anchor (Fig. 1A).

### Vpu stabilizes human tetherin in COS cells

**Figure 2:**
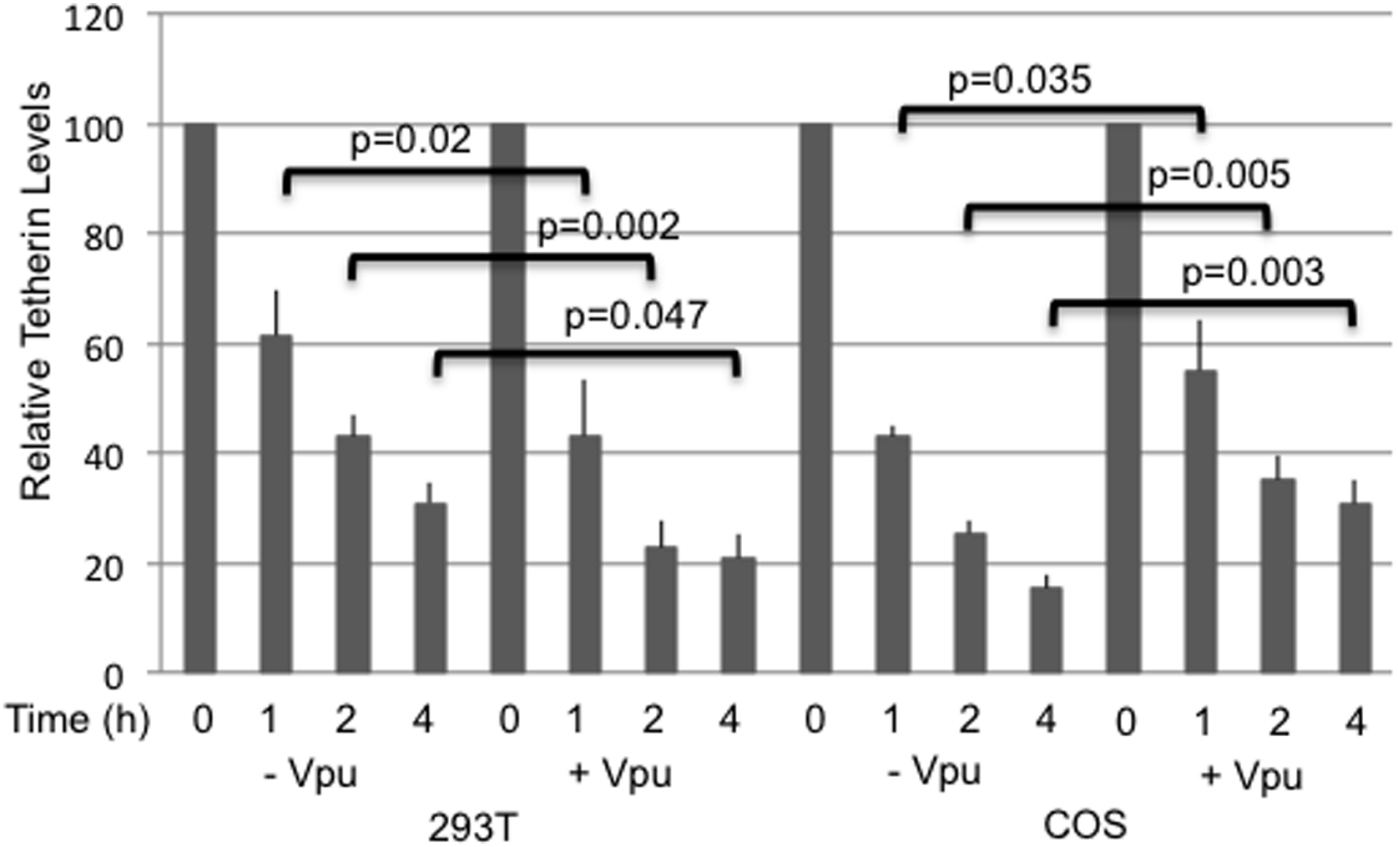
Pulse-chase analysis of tetherin. 293T and COS cells were transfected with HA-tagged human tetherin in the absence and presence of Vpu, 24 h post-transfection cells were labeled with [35S]Met/Cys for 30 min and then chased for 1, 2, or 4 h. Cells were lysed and immunoprecipitated with anti-HA antibodies and analyzed by SDS-PAGE followed by fluorography. Data shown are means ± SD from 4–6 independent experiments.

To investigate whether the Vpu-mediated degradation of human tetherin is cell type-specific, we performed experiments similar to those described above in COS cells, an agm kidney cell line. We have chosen COS cells as they are widely used in cellular and molecular biology research [61] and are often used in HIV assembly studies [62]–[65]. We observed that HA-tagged human tetherin is poorly expressed in COS cells, but, surprisingly, its expression levels are markedly increased upon coexpression with Vpu (Fig. 1B). The deletion mutant delCT is also poorly expressed in COS cells but shows a modest increase in the presence of Vpu. A smaller increase in the expression of the delCT mutant was also observed in 293T cells in the presence of Vpu; the reason for this increase is not clear. The delGPI mutant and mouse, rh, and agm tetherin were highly expressed with or without Vpu (Fig. 1B). To investigate whether the lower expression of human tetherin in COS cells was due to its rapid turnover, we treated COS cells expressing human tetherin with proteasomal (MG132 and ALLN) or lysosomal (bafilomycin and concanamycin) inhibitors. As shown in Fig. 1C, proteasomal inhibitors rescued the expression of the ∼23 kDa tetherin species, whereas lysosomal inhibitors rescued the expression of the ∼26 kDa tetherin species, and, to a lesser extent, the ∼23 kDa species. The stabilization pattern obtained in the presence of Vpu most closely resembled that observed in the presence of lysosomal inhibitors (+Vpu lane, Fig. 1C). These results demonstrate that human tetherin undergoes degradation in COS cells by both proteasomal and lysosomal pathways, with perhaps a greater contribution from the lysosomal pathway. To investigate whether the stabilization of tetherin by Vpu is specific to COS cells we tested in Vero cells, another cell line derived from agm kidney and used in HIV-1 assembly studies [66], [67]. 293T, COS, and Vero cells were transfected with the vector expressing human tetherin without or with Vpu expression vector. Although transfection efficiency is lower in Vero cells, tetherin expression was stabilized by Vpu in Vero cells as in COS cells (Fig. 1D). In 293T cells, as observed before Vpu reduced tetherin expression. To investigate the rate of tetherin turnover in 293T and COS cells we performed pulse-chase analysis. 293T and COS cells were transfected in parallel with tetherin expression vector in the absence and presence of Vpu, pulse-labeled for 30 min and chased for 1, 2, or 4 h. In 293T cells Vpu significantly reduced tetherin levels within 1 h, whereas in COS cells the levels significantly increased compared to those of in the absence of Vpu (Fig. 2).

### The low expression of human tetherin in COS cells depends upon the TM domain

**Figure 3:**
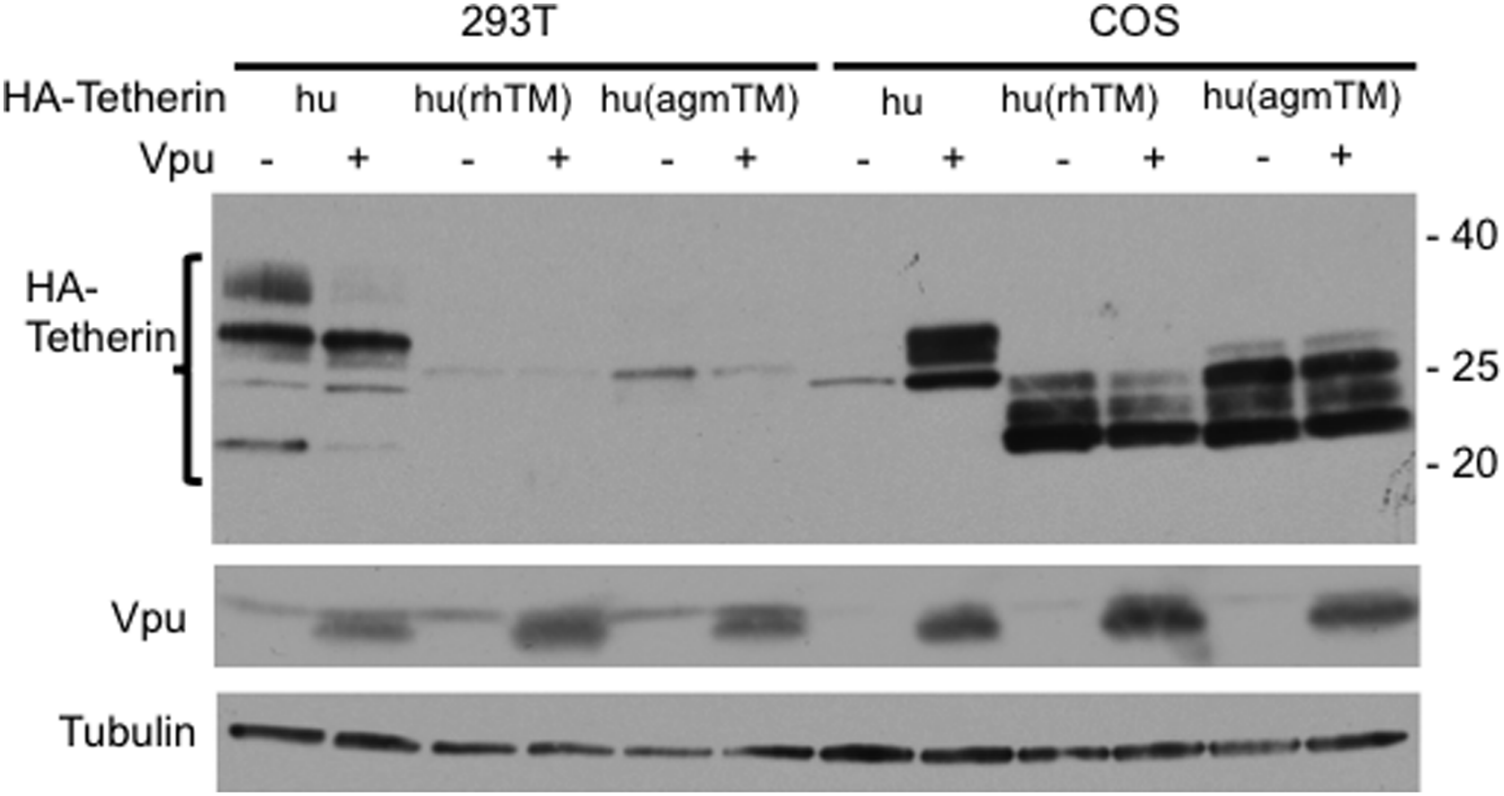
The TM domain of human tetherin is required for its rapid degradation in COS cells. 293T and COS cells were transfected with human (hu) or chimeric tetherins that carry the TM sequences from rh or agm tetherin [hu(rhTM) and hu(agmTM), respectively in the absence or presence of Vpu (1∶5 DNA ratio). One day posttransfection, cell lysates were subjected to western blot analysis with the indicated antibodies. Vpu decreased tetherin expression by 3.0-fold in 293T cells, whereas the levels were increased by 4.5-fold in COS cells. Molecular mass markers are shown on the right (in kDa).

To investigate a possible role for the TM domain of human tetherin in Vpu-mediated stabilization in COS cells, we expressed chimeric human tetherins that carry the TM sequences from rh or agm tetherin [hu(rhTM) and hu(agmTM)] [25]. We observed that these chimeric tetherins are well expressed in COS cells and their expression is not up-regulated by Vpu (Fig. 3). In contrast, these chimeric tetherins are poorly expressed in 293T cells. These results indicate that the TM domain of tetherin influences its expression levels in a cell type-dependent manner and suggest that Vpu binding to the TM domain of human tetherin protects it from rapid degradation in COS cells.

### Vpu counteracts virus release inhibition mediated by human tetherin in COS cells

**Figure 4:**
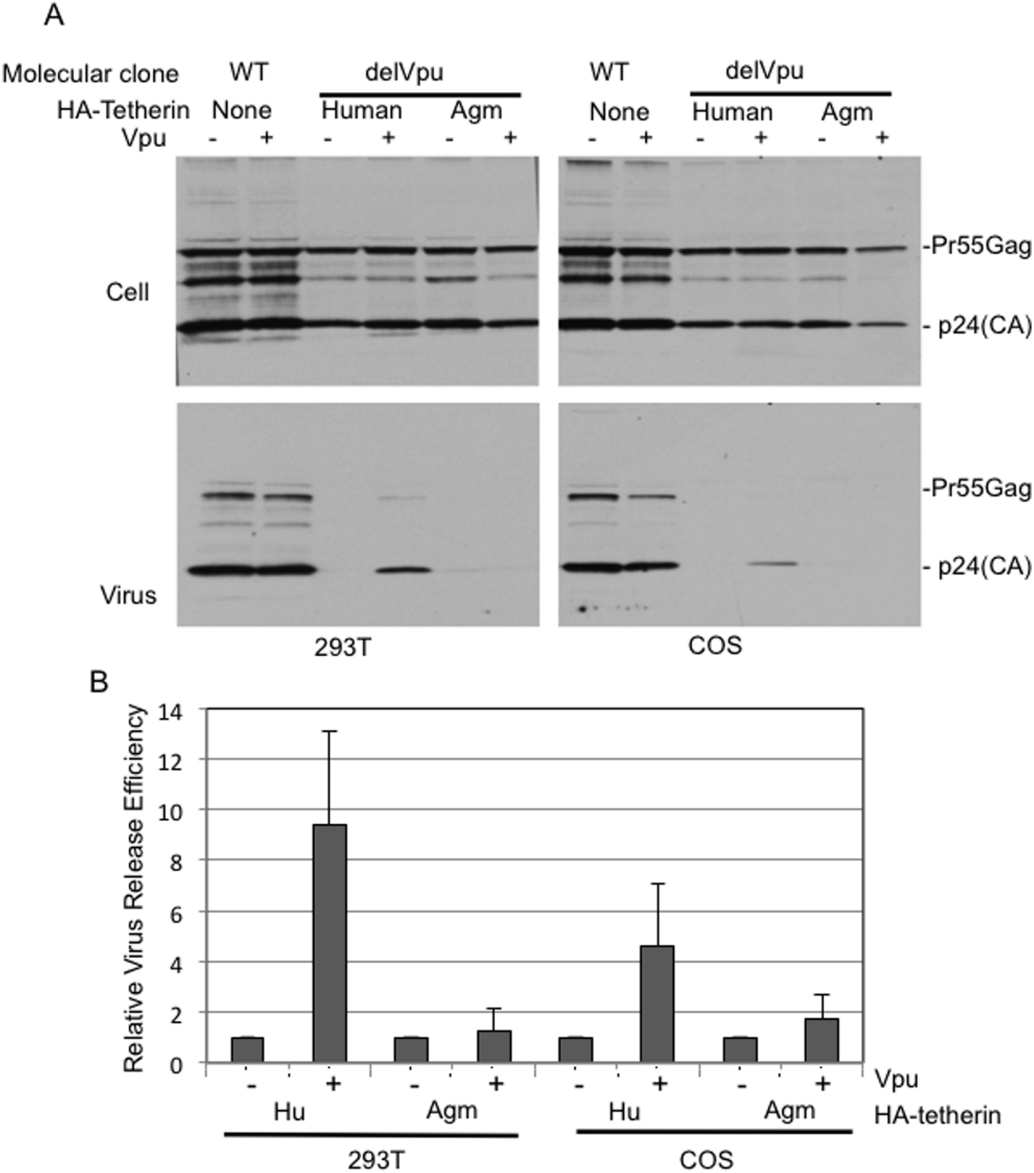
Vpu counteracts the virus release inhibition mediated by tetherin in COS cells. 293T or COS cells were co-transfected with WT pNL4-3 or Vpu-defective (pNL4-3/delVpu) molecular clones and human or agm tetherin expression vectors in the absence or presence of Vpu expression plasmid (15∶1∶5 NL4-3:tetherin:Vpu DNA ratio). (A) One day posttransfection, virus-containing supernatants were harvested and virions were pelleted by ultracentrifugation. Cell and virus lysates were subjected to western blot analysis with HIV-Ig. (B) One day posttransfection with pNL4-3/delVpu without (−Vpu) or with (+Vpu) Vpu expression plasmid and either human or agm tetherin expression vector, cells were metabolically labeled for 2 h, HIV proteins from cell and virus lysates were immunoprecipitated with HIV-Ig and analyzed by SDS-PAGE followed by fluorography. Relative virus release efficiency was calculated as the amount of virion-associated p24 relative to total Gag (cell + virion), normalized to 1 for release in the absence of Vpu. Data shown are means ± SD from four independent experiments.

To examine the impact of tetherin stabilization on the ability of Vpu to counteract tetherin-mediated restriction of virus release, we expressed NL4-3delVpu with human or agm tetherin in the absence or presence of Vpu and measured virus release efficiency by radio-immunoprecipitation analysis. 293T cells were examined in parallel. As reported [1], [68], the release efficiency of delVpu virus is comparable to that of the WT in both 293T and COS cells, as these cell lines are deficient for tetherin expression. In contrast, in the presence of human or agm tetherin, HIV-1 release is severely inhibited in both of these cell lines (Fig. 4A and Fig. S2). Vpu co-expression rescued HIV-1 release by ∼10-fold in 293T cells (Fig. 4B). In COS cells, although Vpu increased the steady-state levels of tetherin (Fig. 1B) it was still able to rescue HIV-1 release by ∼5-fold. As expected [26], Vpu showed minimal effect on virus release in the presence of agm tetherin independent of cell line (Fig. 4B and Fig. S2).

### Cell-surface expression of human tetherin is modestly reduced by Vpu in COS cells

**Figure 5:**
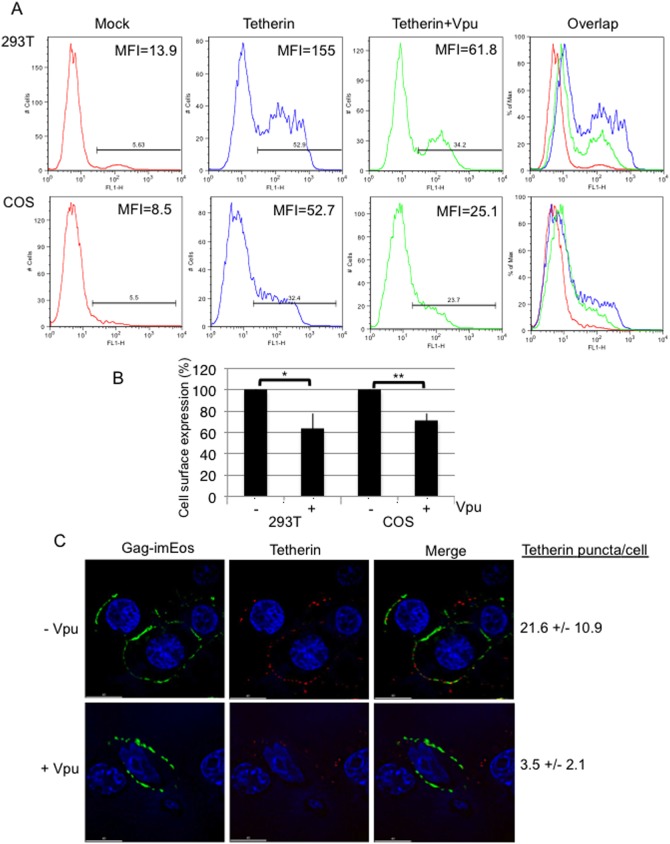
Vpu modestly down-regulates cell-surface expression of tetherin in 293T and COS cells. (A) 293T and COS cells were transfected with vectors expressing human tetherin alone or with the Vpu expression plasmid (1∶5 DNA ratio). Twenty-four h posttransfection, cells were stained with anti-tetherin Ab and cell surface expression was analyzed by flow cytometry and mean fluorescent intensity (MFI) of tetherin-positive cells was determined. (B) Percent of cells positive for tetherin expression in the absence (−) or presence (+) of Vpu. Data from three experiments are shown, ±SD. P values were calculated using the two-tailed unpaired t-test. * = 0.02, ** = 0.002. (C) COS cells were cotransfected with pNL4-3Gag-imEosFPΔPolΔEnv and human tetherin plasmid without or with Vpu (15∶1∶5 NL4-3:tetherin:Vpu DNA ratio). Twenty-four h post-transfection, cell surface tetherin was stained with anti-tetherin Ab prior to fixation with formaldehyde and images were acquired with a Delta-Vision RT deconvolution microscope. 10–15 cells were scored to quantify cell-surface tetherin expression. Scale bar represents 15 µm.

The results presented above raise the possibility that although Vpu increases overall human tetherin expression in COS cells it reduces tetherin levels at sites of virus assembly. We therefore examined the effect of Vpu expression on cell-surface levels of tetherin by flow cytometry using a polyclonal antiserum specific for the ectodomain of tetherin [39]. Again, 293T cells were included for comparison. The mean fluorescent intensity (MFI) of tetherin was reduced by 2.9-fold in 293T and 2.7-fold in COS cells by coexpression of Vpu (Fig. 5A). Further, Vpu coexpression reduced the number of cells expressing detectable levels of tetherin on average by ∼36% in 293T and ∼29% in COS cells (Fig. 5B). As an independent means of measuring cell-surface tetherin expression, we analyzed PM localization of tetherin by immunofluorescence and also measured colocalization between Gag and tetherin by co-expressing Gag containing a monomeric, photoactivatable fluorescent protein (mEos) inserted between the MA and CA domains (Gag-imEos). As previously reported [10], in the presence of Gag, tetherin showed a punctate staining on the cell surface that partially overlapped with the Gag localization pattern. The number of puncta was scored and expressed as the average number of puncta per cell. As shown in Fig. 5C, the number of tetherin puncta (counted from 10–15 cells) on the cell surface (red) of COS cells is markedly higher in the absence of Vpu (21.6±10.9) than in its presence (3.5±2.1). Together, these results indicate that although the expression levels of human tetherin in COS cells are upregulated by Vpu the amount of tetherin on the cell surface is reduced in the presence of Vpu.

### Vpu co-expression results in intracellular sequestration of human tetherin in COS cells

**Figure 6:**
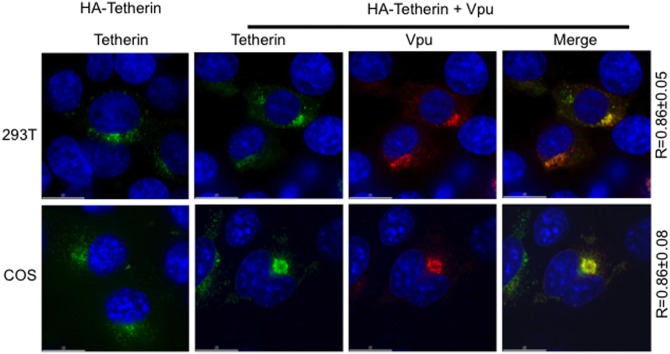
Vpu induces the sequestration of tetherin in COS cells. 293T and COS cells were transfected with vector expressing HA-tagged tetherin alone or in combination with Vpu expression vector (1∶5 DNA ratio). Twenty-four h post-transfection, cells were fixed with 4% formaldehyde, permeabilized with methanol and stained with anti-HA (green), anti-Vpu (red), or DAPI (blue) and were analyzed with a Delta-Vision RT deconvolution microscope. Numbers represent the Pearson correlation coefficient (R) ± SD from 10–15 cells. Scale bars, 15 µm.

We next investigated the localization of tetherin in Vpu-expressing COS cells. Human tetherin was expressed in 293T and COS cells in the absence or presence of Vpu, and transfected cells were stained with anti-HA and anti-Vpu antibodies. As shown in Fig. 6, in 293T cells human tetherin is localized both on the cell surface and in intracellular compartments. Vpu colocalizes with human tetherin to a significant extent (Pearson correlation coefficient R = 0.86±0.05). In COS cells, the Vpu–tetherin colocalization values are essentially the same (R = 0.86±0.08) as those in 293T cells. However, in COS cells, Vpu sequesters human tetherin into intracellular vesicular compartments in the cytosol (Fig. 6). To investigate time course of Vpu-induced subcellular distribution of tetherin in 293T and COS cells we fixed the cells at time points 12, 18, and 24 h post-transfection and examined tetherin localization. In COS cells Vpu induced the formation of intracellular vesicular compartments; these are seen as early as 12 h and continuously grow at 18 h and form vesicular structures at 24 post-transfection (Fig. S3B). In 293T cells Vpu decreases tetherin levels as early as 18 h post-transfection (Fig. S3A).

### Tetherin colocalizes with lysosomal markers in COS cells in the presence of Vpu

**Figure 7:**
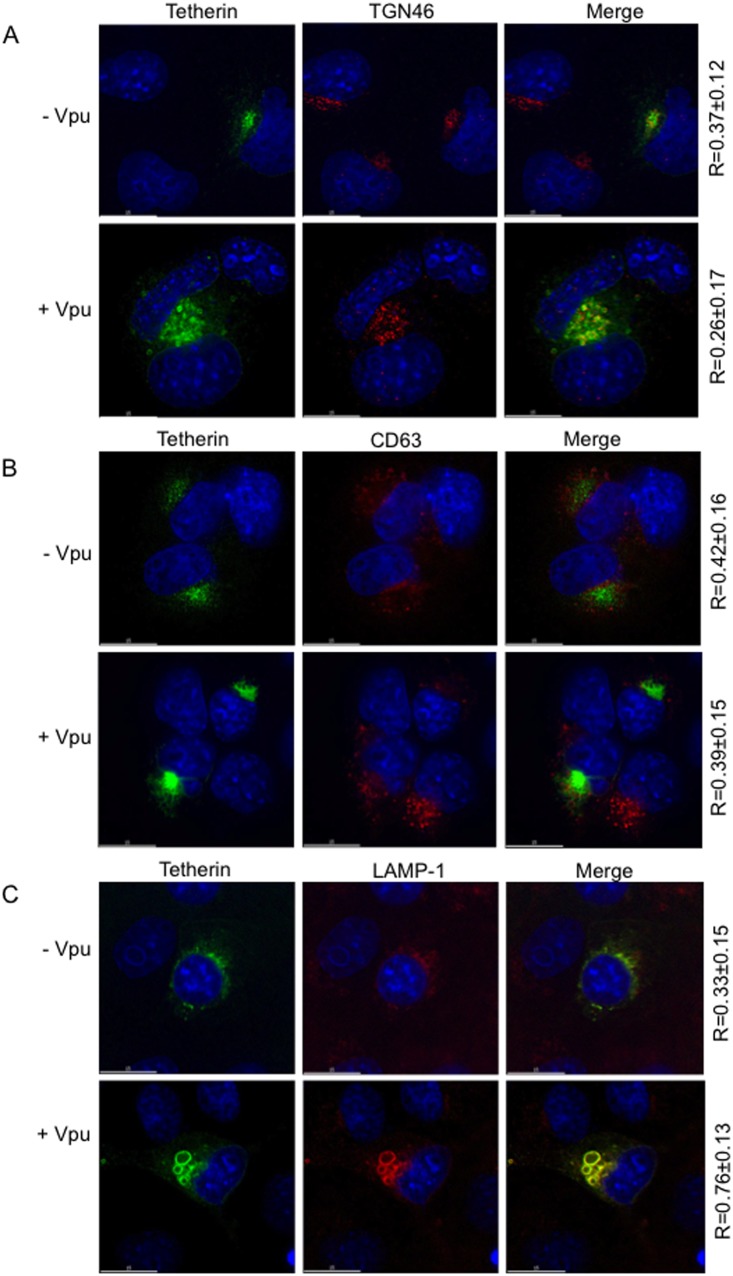
Tetherin colocalizes with lysosomal markers in COS cells in the presence of Vpu. COS cells were transfected with HA-tagged tetherin expression vector alone or in combination with Vpu expression vector (1∶5 DNA ratio). Twenty-four h post-transfection, cells were fixed and permeabilized as in Fig. 6 and stained with anti-HA (green), DAPI (blue), and (A) the TGN marker TGN46 (red), (B) the multivesicular body marker CD63 (red), or (C) the lysosomal marker LAMP-1 (red). Images were acquired with a DeltaVision RT deconvolution microscope. Numbers represent the Pearson correlation coefficient (R) ± SD from 10–20 cells. Scale bars, 15 µm.

We next characterized the compartments in which tetherin is sequestered by Vpu in COS cells. Cells expressing tetherin in the absence or presence of Vpu were stained for tetherin and the TGN marker TGN46 or the late endosome marker CD63. Co-localization values for tetherin and TGN46 were 0.37±0.12 and 0.26±0.17 in the absence and presence of Vpu, respectively (Fig. 7A). The R-values for tetherin and CD63 were 0.42±0.16 and 0.39±0.15 in the absence and presence of Vpu, respectively (Fig. 7B). These relatively low colocalization values suggest that tetherin is not sequestered in the TGN or in late endosomes by Vpu in COS cells. We then stained with the lysosomal marker LAMP-1 (lysosomal-associated membrane protein 1). The colocalization values were significantly higher in the presence of Vpu (0.76±0.13) than in its absence (0.33±0.15) (Fig. 7C). These results indicate that in COS cells Vpu sequesters tetherin in a compartment that is positive for lysosomal markers. As shown earlier (Fig. 1C), treating COS cells with lysosomal inhibitors in the absence of Vpu resulted in the stabilization of tetherin to a similar extent as observed in the presence of Vpu. We therefore investigated whether adding lysosomal inhibitors to tetherin-transfected COS cells leads to tetherin accumulation in LAMP-1-positive compartments. Indeed, treating COS cells with concanamycin or bafilomycin results in the accumulation of tetherin in LAMP-1-positive compartments even in the absence of Vpu (Fig. S4). In the presence of Vpu, lysosomal inhibitors have little or no additional effect on the co-localization of tetherin with LAMP-1 (Fig. S4). The fact that in COS cells Vpu induces the accumulation of tetherin in a LAMP-1-positive compartment without inducing tetherin degradation suggests that the LAMP-1-positive compartment in which tetherin accumulates in COS cells represents an aberrant, non-functional lysosomal compartment.

## Discussion

In this study, we analyzed the relationship between Vpu-mediated degradation of tetherin and enhancement of virus release. Our results demonstrate that Vpu degrades tetherin in 293T cells by both proteasomal and lysosomal pathways, and that reduced levels of tetherin are associated with Vpu-mediated enhancement of virus release in this cell line (Fig. 4). In contrast, we find that in COS and Vero cells, human tetherin is poorly expressed in the absence of Vpu but its expression is stabilized in the presence of Vpu. Nonetheless, despite the fact that human tetherin levels are higher in the presence of Vpu, virus release is still enhanced by Vpu in COS cells. These results add to a growing body of evidence indicating that Vpu can overcome the antiviral activity of tetherin without inducing its degradation. For example, Strebel and co-workers reported that during the course of productive infection in T cells Vpu enhances HIV-1 release without reducing tetherin’s cell surface or intracellular expression [39]. Goffinet et al. observed that Vpu is able to induce a small increase in the expression of a tetherin mutant (K18, 21A) in 293T cells while still antagonizing its antiviral activity [38]. In our study, although Vpu enhanced the total expression of human tetherin in COS cells, the cell-surface expression was modestly reduced by Vpu. These results are consistent with observations from others that Vpu-induced reductions in cell-surface tetherin expression correlate with loss of antiviral activity, but is uncoupled from total cellular levels of tetherin [27], [32], [35], [38], [40].

The molecular determinants in human tetherin for Vpu antagonism are reported to reside in the TM domain, with specific residues apparently regulating direct Vpu–tetherin interactions [25], [26], [33], [35], [40], [52], [69], [70]. The inability of Vpu to antagonize non-human tetherin correlates with a lack of interaction with Vpu [17], [26], [27]. The interaction of Vpu with human tetherin likely plays a role in Vpu-mediated tetherin stabilization in COS cells, as Vpu has no significant effect on the expression of chimeric tetherins that carry the TM domain from rh or agm tetherin. Thus, we speculate that Vpu binding to human tetherin protects it from rapid degradation in COS cells. Moreover, the TM-chimeric tetherins were well expressed in COS cells but poorly expressed in 293T cells, indicating that the TM domain of tetherin regulates its cell-type dependent stability.

We observed that in COS cells Vpu sequesters human tetherin in a compartment that bears markers for lysosomes (e.g., LAMP-1) but shows little overlap with multivesicular body (CD63) or TGN (TGN46) markers. Earlier studies showed sequestration of tetherin in the TGN induced by Vpu [14], [71], HIV-2 Env [14], [15], and SIV Env [13]. Tetherin accumulation in perinuclear patches was also observed to be induced by Lassa virus Z protein, but the nature of these compartments was not characterized [11]. It is intriguing that the increased colocalization of tetherin with LAMP-1 that we observe in COS cells in the presence of Vpu results in the stabilization rather than the degradation of tetherin. We speculate that Vpu expression in COS cells results in the generation of an aberrant lysosomal compartment, as suggested by the swollen, ring-like morphology of these structures (Fig. 7). Accumulation of tetherin in a LAMP-1-positive compartment in COS cells treated with lysosomal inhibitors even in the absence of Vpu is consistent with the hypothesis that Vpu induces formation of aberrant lysosomal compartments in which tetherin accumulates. This could provide HIV-1 with a Vpu-dependent mechanism for disrupting lysosomal function, thereby preventing the degradation of virally encoded proteins. While this hypothesis will require further testing, the results of this study demonstrate that COS cells provide a system for the molecular dissection of Vpu and tetherin activities.

## Supporting Information

Figure S1 **Vpu mediates proteasomal and lysosomal degradation of tetherin.** (A) 293T cells were transfected with empty vector alone or HA-tagged tetherin expression plasmid without or with combination of Vpu or the indicated proviral expression plasmid (1∶5 DNA ratio). One day posttransfection, cells were lysed and subjected to SDS-PAGE and subsequent western blot analysis with anti-HA (top panel) or anti-tubulin (lower panel) antibodies. Levels of tetherin expression were reduced in the presence of Vpu by approximately three-fold in this and repeat experiments, as determined by band quantification with an Alpha Innotech Fluorchem SP imager. Vpu-defective molecular clones, delVpu and Udel did not reduce tetherin levels. Molecular mass markers are shown on the right (in kDa). (B) 293T cells were transfected with HA-tagged tetherin expression vector alone (left panel; −Vpu) or in combination with the Vpu expression plasmid (right panel; +Vpu). One day posttransfection, cells were treated with proteasomal and lysosomal inhibitors at the following concentrations: MG132 (25 µM), ALLN (25 µM), bafilomycin (0.15 µM) and concanamycin (0.5 µM). Levels of HA-tagged tetherin and tubulin were determined by western blotting with anti-HA and anti-tubulin antibodies, respectively. Molecular mass markers are shown on the right (in kDa). Treating Vpu and tetherin-expressing cells with proteasomal inhibitors enhanced the expression of tetherin approximately six-fold. Lysosomal inhibitors increased expression of the ∼26 kDa band by approximately four-fold, whereas the highly glycosylated forms of tetherin (∼30–36 kDa) were not recovered significantly.
(TIFF)

Figure S2 **293T or COS cells were co-transfected with molecular clones (pNL4-3 or pNL4-3/delVpu) and human or agm tetherin expression vectors in the absence or presence of Vpu expression plasmid as in** **Fig. 4A** **.** One day posttransfection, virus supernatants were collected and RT activity was measured. Data shown are means ± SD from two independent experiments.
(TIFF)

Figure S3 **293T and COS cells were transfected with vectors expressing human tetherin alone or with the Vpu expression plasmid (1∶5 DNA ratio) and fixed after 12, 18, and 24 h post-transfection with 4% formaldehyde.** Cells were then permeabilized and stained with anti-tetherin Ab as in Fig. 6. Images shown are representative from 8–10 cells. Scale bars, 15 µm.
(TIFF)

Figure S4 **COS cells were transfected with HA-tagged tetherin expression vector in the absence or presence of Vpu (1∶5 DNA ratio).** One day post-transfection, cells were treated with lysosomal inhibitors concanamycin (0.5 µM) and bafilomycin (0.15 µM), fixed, permeabilized as in Fig. 6 and stained with anti-HA (green), DAPI (blue), and LAMP-1 (red). Numbers represent the Pearson correlation coefficient (R) ± SD from 10–12 cells. Scale bars, 15 µm.
(TIFF)
