# Supplementary figures and images for: In COS Cells Vpu Can Both Stabilize Tetherin Expression and Counteract Its Antiviral Activity
Source: PLoS One. 2014 Oct 31;9(10):e111628. doi: 10.1371/journal.pone.0111628 (PMC4216104; doi:10.1371/journal.pone.0111628)

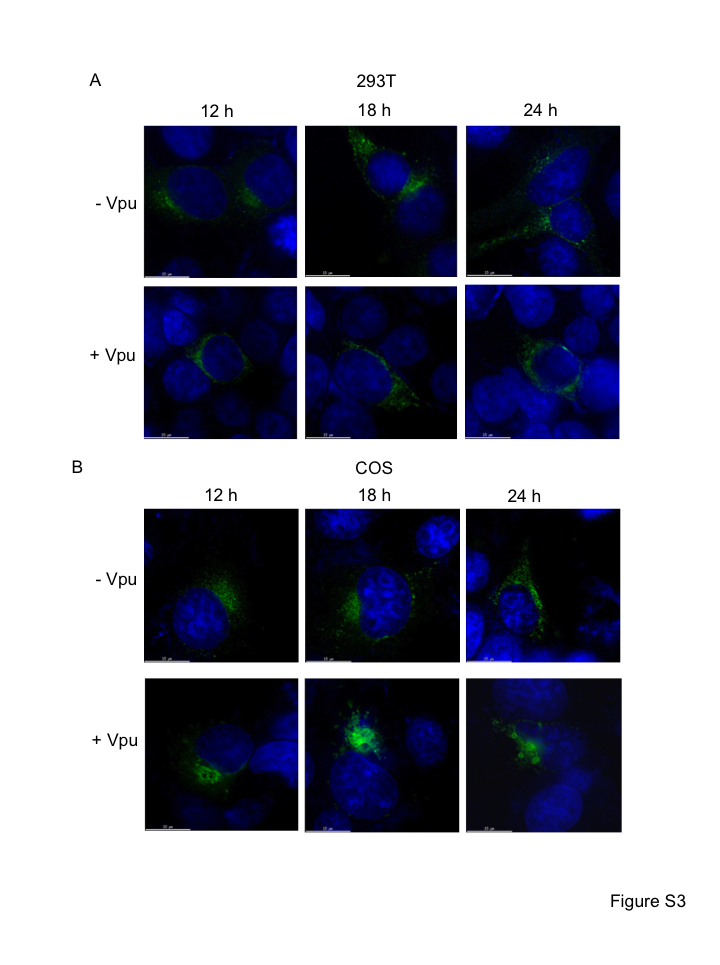

Supplement: Figure S3 — 293T and COS cells were transfected with vectors expressing human tetherin alone or with the Vpu expression plasmid (1∶5 DNA ratio) and fixed after 12, 18, and 24 h post-transfection with 4% formaldehyde. Cells were then permeabilized and stained with anti-tetherin Ab as in Fig. 6. Images shown are representative from 8–10 cells. Scale bars, 15 µm. (TIFF) [file pone.0111628.s003.tiff]

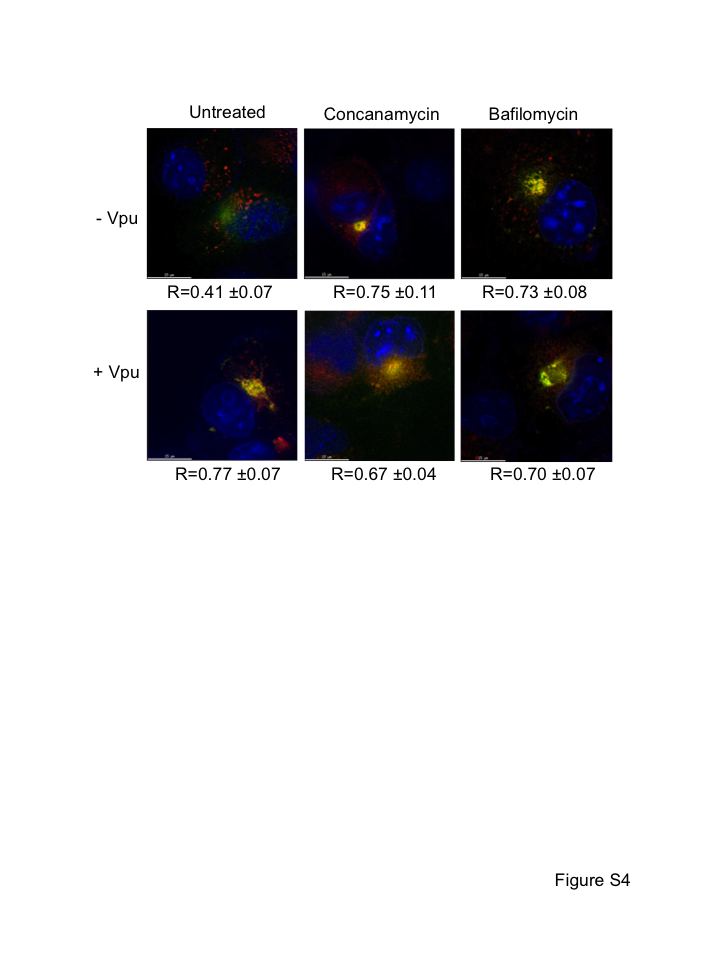

Supplement: Figure S4 — COS cells were transfected with HA-tagged tetherin expression vector in the absence or presence of Vpu (1∶5 DNA ratio). One day post-transfection, cells were treated with lysosomal inhibitors concanamycin (0.5 µM) and bafilomycin (0.15 µM), fixed, permeabilized as in Fig. 6 and stained with anti-HA (green), DAPI (blue), and LAMP-1 (red). Numbers represent the Pearson correlation coefficient (R) ± SD from 10–12 cells. Scale bars, 15 µm. (TIFF) [file pone.0111628.s004.tiff]
